# Prognostic role of B7-H4 in patients with non-small cell lung cancer: A meta-analysis

**DOI:** 10.18632/oncotarget.15648

**Published:** 2017-02-23

**Authors:** Zhibo Tan, Weixi Shen

**Affiliations:** ^1^ Department of Oncology, Shenzhen Hospital of Southern Medical University, Shenzhen, Guangdong, P.R. China

**Keywords:** meta-analysis, B7-H4, lung cancer, evidence-based medicine

## Abstract

B7 homolog 4 (B7-H4) has been recently reported to be a prognostic marker in non-small cell lung cancer (NSCLC) in some studies. However, the results remained conflicting. Thus, we aimed to comprehensively assess the association between B7-H4 expression and prognosis of NSCLC patients by performing a meta-analysis. Relevant publications were thoroughly searched of PubMed, Embase, Web of Science and China National Knowledge Infrastructure (CNKI). The pooled odds ratios (ORs) and hazard ratios (HRs) with 95% confidence intervals (CIs) were applied to evaluate the effects. A total of 9 studies comprising 1444 patients were included in this meta-analysis. B7-H4 overexpression was associated with presence of lymph node metastasis (OR=3.59, 95%CI=2.39-5.38, p<0.001; fixed effect), advanced TNM stage (OR=2.36, 95%CI=1.2-4.67, p=0.013; random effect), and poor differentiation (OR=2.11, 95%CI=1.12-3.99, p=0.021; fixed effect). However, B7-H4 had no significant correlation with gender, age or histology in NSCLC. Furthermore, in a fixed effects model, the results indicated that B7-H4 overexpression was significantly associated with poor OS (HR=2.03, 95%CI=1.41-2.92, p<0.001). This meta-analysis demonstrated that high B7-H4 expression is an unfavorable prognostic factor in NSCLC. Because few studies were included for meta-analysis and almost all included studies were performed on Chinese patients, therefore; large scale prospective studies are needed to verify our results.

## INTRODUCTION

Lung cancer is the most commonly diagnosed cancer around the world [1]. The prognosis of lung cancer is also poor, and the overall 5 year survival is about 15% over the past decades [2]. Non-small cell lung cancer (NSCLC), accounting for approximately 80%-85% of all lung cancer cases, is the main form of lung cancer [3]. More than half of NSCLC patients are in advanced stages (stage IIIB/IV) at first diagnosis, leading to a poor prognosis [2]. Despite several prognostic markers including performance status (PS), age and weight loss have been identified for prognostication. The predicative accuracy is still unsatisfactory [4]. Obviously, more reliable prognostic markers are needed to make the management of NSCLC be more individualized.

Immune escape is recognized as an important characteristic of cancer cells [5]. B7 homolog 4 (B7-H4, B7S1, B7x) is a member of the B7 family. Members of the B7 family are involved in immune escape of tumor cells, since the level of activation of the anti-tumor immune response depends on the balance between co-stimulatory and co-inhibitory signals [6]. B7-H4 shares about 25% amino acid homology with other B7 family members including B7-H1 (PD-L1), B7-DC (PD-L2) and B7-H3 [7, 8]. B7-H4 palys a pivotal role in the regulation of tumor microenvironment. Evidence shows that B7-H4 could act as a co-inhibitor of T-cell response and innate immunity [9, 10]. B7-H4 expression was also investigated as a potential prognostic marker in a variety of solid tumors including osteosarcoma, NSCLC, oral squamous cell carcinoma, hepatocellular carcinoma, gastric cancer and glioma [11–18]. A number of studies have explored the prognostic significance of B7-H4 in NSCLC [16–27], whereas the results were controversial. Li *et al*. reported that in NSCLC patients with brain metastases, patients with high B7-H4 expression survived shorter than patients with low B7-H4 expression (p= 0.002)[23]. Furthermore, Wang and colleagues also reported that B7-H4 serum level was an independent prognostic indicator of overall survival (OS) and progression-free survival (PFS) (p<0.01)[26]. However, Xu *et al*. showed that B7-H4 expression was not significantly associated with OS in NSCLC (p=0.32)[22]. Therefore, we performed a meta-analysis of published data to more precisely assess the impact of B7-H4 on prognosis of NSCLC.

## RESULTS

### Studies selection and characteristics

**Figure 1:**
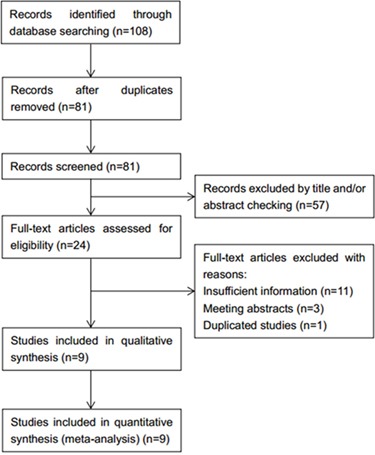
Flow diagram of the inclusion and exclusion of studies

One hundred and eight potentially relevant records were obtained after initial search (Figure 1). After duplicates were excluded, 81 records were screened by title and/or abstract and 57 records were subsequently removed. A total of 24 studies were evaluated for eligibility by full-text screening. Then, 15 studies were excluded due to the following reasons: insufficient data (n=11), meeting abstracts (n=3), and duplicate studies (n=1). Finally, 9 eligible studies [16–18, 22–27] were included for meta-analysis. The flow diagram shows the literature selection process (Figure 1). The main characteristics of the 9 included studies were shown in Table 1. The total sample was 1444, ranging from 49 to 552 patients per study. Eight studies [16–18, 22–24, 26, 27] were conducted in China and one study [25] was performed in USA. Five studies [16, 23, 25–27] were in English and 4 studies [17, 18, 22, 24] were in Chinese. All studies were assigned with NOS scores more than 6.

**Table 1:** Main characteristic of included studies

First author	Year	NOS score	Country	Sample size	Gender (M/F)	Female (%)	Detection method	Tumor stage
Sun [16]	2006	7	China	70	49/21	30	IHC	I-III
Zhang [17]	2009	6	China	52	30/22	42.3	IHC	I-IV
Qin [18]	2010	7	China	80	52/28	35	IHC	I-III
Xu [22]	2011	7	China	103	67/36	35	IHC	I-IV
Li [23]	2013	7	China	49	37/12	24.5	IHC	IV
Liu [24]	2015	6	China	94	44/50	53.2	IHC	NR
Schalper [25]	2016	7	USA	552	316/236	42.8	QIF	I-IV
Wang [26]	2016	8	China	316	160/156	49.4	ELISA	I-IV
Xu [27]	2016	7	China	128	80/48	37.5	ELISA	I-IV

Abbreviations: IHC: immunohistochemistry; QIF: quantitative immunofluorescence; ELISA: enzyme linked immunosorbent assay; NR: not reported.

### Correlation of B7-H4 with clinicopathological parameters

**Figure 2:**
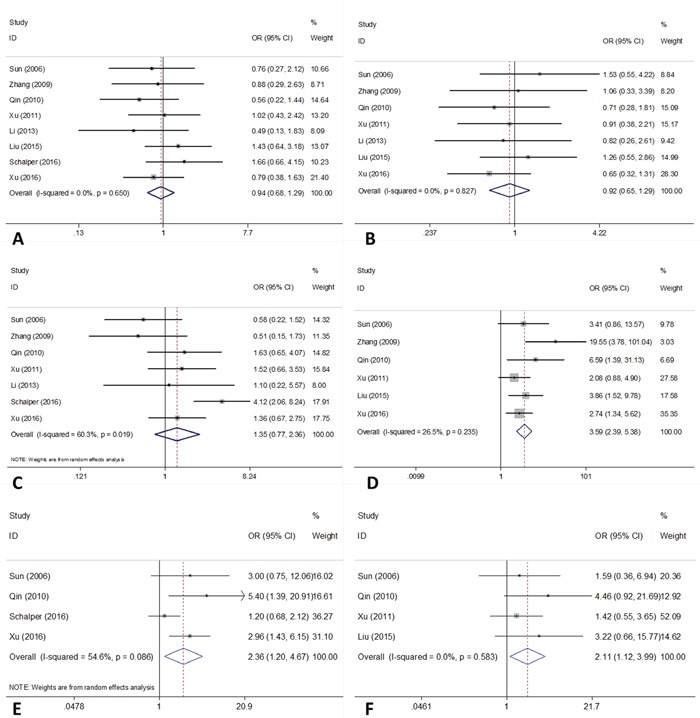
Forest plots of OR for the relation between B7-H4 expression and **(A)** gender; **(B)** age; **(C)** histology; **(D)** lymph node metastasis; **(E)** TNM stage and **(F)** differentiation.

The main results showing the association of B7-H4 with clinicalpathological factors were summarized in Table 2 and Figure 2. Six studies [16–18, 22, 24, 27] reported the correlation between B7-H4 and lymph node metastasis. The pooled data were: OR=3.59, 95%CI=2.39-5.38, p<0.001, (fixed effect) suggesting that B7-H4 overexpression was associated with presence of lymph node metastasis in NSCLC. In addition, pooled data from 4 studies [16, 18, 25, 27] also suggested high B7-H4 expression was correlated with advanced TNM stage (OR=2.36, 95%CI=1.2-4.67, p=0.013; random effect). Four studies [16, 18, 22, 24] reported the data on differentiation, the pooled results (OR=2.11, 95%CI=1.12-3.99, p=0.021; fixed effect) showed that B7-H4 expression was also associated with poor differentiation. However, the pooled data from 8 studies [16–18, 22–25, 27], 7 studies [16–18, 22–24, 27] and 7 studies [16–18, 22, 23, 25, 27] suggested that there was no significant association of B7-H4 with gender, age or histology in NSCLC (Table 2, Figure 2).

**Table 2:** Association between B7-H4 and clinicalpathological factors in NSCLC

Variables	No. of studies	No. of patients	Effects model	OR (95%CI)	p	Heterogeneity	
						*I*^2^(%)	Ph
Gender (male vs female)	8	1,128	Fixed	0.94(0.68-1.29)	0.699	0	0.65
Age (≥60 vs <60, years)	7	576	Fixed	0.92(0.65-1.29)	0.617	0	0.827
Histology (SCC vs ADC)	7	1,034	Random	2.35(0.77-2.36)	0.289	60.3	0.019
Lymph node metastasis (yes vs no)	6	527	Fixed	3.59(2.39-5.38)	<0.001	26.5	0.235
TNM stage (III+IV vs I+II)	4	830	Random	2.36(1.2-4.67)	0.013	54.6	0.086
Differentiation (poor vs moderate/well)	4	347	Fixed	2.11(1.12-3.99)	0.021	0	0.583

Abbreviations: SCC: Squamous cell carcinoma; ADC: Adenocarcinoma

### Impact of B7-H4 expression on OS of NSCLC

**Figure 3:**
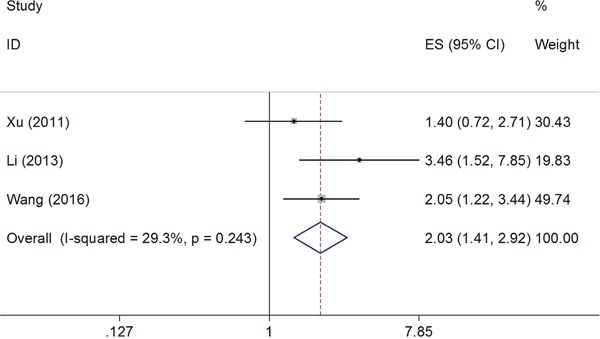
Forest plot for the association of B7-H4 expression with overall survival

The HRs for OS were available in 3 studies [22, 23, 26] involving 468 patients. As the heterogeneity among studies was not significant (*I*^2^=29.3%, P_h_=0.243), the fixed effects model was used (Figure 3). The pooled HR showed a significant correlation of B7-H4 with shorter OS (HR=2.03, 95%CI=1.41-2.92, p<0.001; Figure 3).

### Publication bias

**Figure 4:**
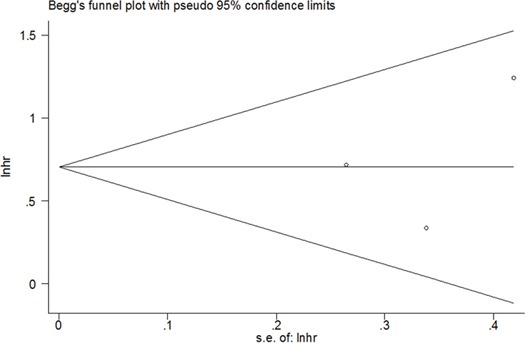
Funnel plot for publication bias assessment

Publication bias was tested using Begg's funnel plot in this meta-analysis. As shown in Figure 4, the funnel plot was symmetrical and showed no evidence of publication bias (Begg's p= 0.602).

## DISCUSSION

To our knowledge, this is the first meta-analysis on the relationship between B7-H4 expression and clinicalpathological features and OS in NSCLC patients. By aggregating data from 9 studies with 1444 patients, we showed that elevated B7-H4 expression was significantly associated with presence of lymph node metastasis, advanced TNM stage, poor differentiation and shorter OS in NSCLC. Taken together, these results suggested that B7-H4 could act as a potential prognostic factor for NSCLC patients.

B7-H4 is a new member of B7 family which was identified in 2003 [28]. B7-H4 had a profound inhibitory effect on development and function of T cells [28]. Recent investigations also showed that B7-H4 overexpression could promote the proliferation of FOXP3+ regulatory T cells (Tregs) and the secretion of IL-10 and TGF-β1 [29]. B7-H4 was also found to be correlated with Tregs infiltration and thus facilitate the immune tolerance in tumor microenvironment [30]. There is also evidence showing that B7-H4 suppresses the function of antigen presenting cells (APCs). Kryczek et al demonstrate that Treg cells trigger high levels of IL-10 production by APCs, stimulate APC B7-H4 expression, and render APCs immunosuppressive [31]. B7-H4 was found to be expressed in various cancers, including gastric cancer [15, 32], renal cell carcinoma [33], pancreatic cancer [34] and prostate cancer [35]. Studies also suggested that B7-H4 was a prognostic indicator of poor survival and different clinicalpathological features in various cancers [32, 34, 35].

We found that several studies [36–38] had investigated the correlation between B7-H4 and prognosis of cancer patients using meta-analysis. Song et al combined data from 18 studies and demonstrated that B7-H4 was significantly associated with worse OS across cancer types. Cui et al [36] found that B7-H4 is predictive of poor prognosis in gastric cancer. These observations were in line with the results on NSCLC in the current meta-analysis. Notably, in the meta-analysis [37] exploring the impact of B7-H4 on prognosis of solid tumors, only one study [23] on NSCLC was included and NSCLC could not be analyzed separately. However, in the current analysis containing 9 studies on NSCLC, we analyzed the association between B7-H4 and clinicalpathological factors and survival outcomes. Therefore, this is the first meta-analysis on the relationship between B7-H4 expression and clinicalpathological features and OS in NSCLC patients. We noted included studies used various methods including immunohistochemistry (IHC), quantitative immunofluorescence (QIF), and enzyme linked immunosorbent assay (ELISA) to detect B7-H4 expression. Thus, the sensitivity of different methods may vary, resulting in inconsistent cut-offs in each study. This issue may lead to conflicting results and have potential impacts on this meta-analysis. Therefore, further studies with uniform detection method are needed.

There were also several limitations to this meta-analysis. First, although we searched literature both in English and Chinese, most included studies were conducted in China. Therefore, patient selection bias maybe exists. Second, the eligible studies for OS analysis were limited. Only 3 studies were included for the OS analysis. More large scale studies are needed for meta-analysis to make the conclusions more concrete.

In summary, despite the limitations listed above, this meta-analysis demonstrated that elevated B7-H4 expression was significantly associated with presence of lymph node metastasis, advanced TNM stage, poor differentiation and shorter OS in NSCLC. High B7-H4 expression is an unfavorable prognostic factor in NSCLC.

## MATERIALS AND METHODS

### Search strategy

We conducted this meta-analysis under the guideline of the Preferred Reporting Items for Systematic Reviews and Meta-Analyses (PRISMA) statement [39]. We performed a comprehensive literature search of the following databases: PubMed, Embase, Web of Science and China National Knowledge Infrastructure (CNKI). The last search was updated on November 10, 2016. The published languages were limited to English and Chinese. The searching items were: “B7-H4”, “B7x”, “B7S1”, “lung cancer”, “lung carcinoma”, “lung neoplasm” and “lung tumor”. The reference lists of the selected articles were searched to identify relevant studies.

### Selection criteria

The inclusion criteria were as follows: (1) diagnosis of NSCLC was proven by histopathological methods; (2) B7-H4 was detected by any method; (3) studies reported the correlation of B7-H4 with clinicopathological factors or overall survival (OS); (4) when there were multiple studies on the same patients population, the largest or the most recent one was included; (5) articles were published in English or Chinese. The exclusion criteria were as follows: (1) meeting abstracts, reviews, letters, or case reports; (2) duplicated studies; (3) studies which lack necessary information.

### Data extraction and quality assessment

Two investigators (ZBT and WXS) independently assessed the articles and extracted the following information form included studies: first author, publication year, study country, sample size, gender distribution, detection methods and tumor stage. The quality of each study was using the Newcastle-Ottawa Quality Assessment Scale (NOS)[40]. A study can be awarded a maximum of 9 points and studies obtained more than 6 were assigned as high quality studies (Table 3). Disagreements between the two investigators were resolved by consensus.

**Table 3:** Quality assessment of the included studies according to the Newcastle–Ottawa scale

Studies	Selection (4 stars in total, 1 for each item)				Comparability (2 stars)	Outcome (3 stars in total, 1 for each item)			Total score
	Representativeness of the exposed cohort	Selection of the nonexposed cohort	Assessment of exposure	Outcome not present at start of study		Assessment of outcome	Follow-up long enough for outcomes	Adequacy of follow-up	
Sun [16]	1	1	1	1	2	1	0	0	7
Zhang [17]	1	1	1	1	2	0	0	0	6
Qin [18]	1	1	1	1	2	1	0	0	7
Xu [22]	1	1	1	1	1	1	1	0	7
Li [23]	1	1	1	1	1	1	1	0	7
Liu [24]	1	1	1	1	1	1	0	0	6
Schalper [25]	1	1	1	1	2	1	0	0	7
Wang [26]	1	1	1	1	1	1	1	1	8
Xu [27]	1	1	1	1	2	1	0	0	7

### Statistical analysis

STATA 12 (StataCorp LP, College Station, TX, USA) was employed to conduct this meta-analysis. Odds ratios (ORs) and their 95% confidence intervals (CIs) were used to estimate the association between B7-H4 expression and clinicopathological factors, including gender, age, histology, lymph node metastasis, TNM stage, and differentiation. Statistical heterogeneity across studies was assessed by using Cochran's Q and *I*^2^ statistics. Heterogeneity was considered as statistically significant at *I*^2^>50% and P_h_<0.10. In that case, the random effect model was used; otherwise, the fixed effect model was applied. Hazard ratios (HRs) and corresponding 95 % CIs were pooled for the analysis of B7-H4 expression and OS. Begg's funnel plot was generated to examine potential publication bias. All statistical tests were two-sided, and a P value <0.05 was considered as statistically significant.
